# Influence of Statin Therapy on the Incidence of Cardiovascular Events, Cancer, and All-Cause Mortality in People Living With HIV: A Meta-Analysis

**DOI:** 10.3389/fmed.2021.769740

**Published:** 2021-11-08

**Authors:** Yanping Li, Zhandi Wang, Haimei Xia, Ju Zhang

**Affiliations:** ^1^School of Chemistry, Biology and Environment, Institute of Biology and Environmental Engineering, Yuxi Normal University, Yuxi, China; ^2^Center of Chinese Medicine, Yunnan Institute of Traditional Chinese Medicine, Kunming, China

**Keywords:** human immunodeficiency virus, statin, mortality, cardiovascular, cancer

## Abstract

**Background:** Possible influences of statin therapy on the risk of cardiovascular events, cancer, and all-cause mortality in people living with HIV (PLWH) remain unclear. We performed a meta-analysis to systematically evaluate the efficacy of statin in PLWH.

**Methods:** Relevant cohort studies were retrieved via a search of the Medline, the Embase, and the Web of Science databases until June 14, 2021. The data were combined with a random-effects model by incorporating the between-study heterogeneity.

**Results:** A total of 12 multivariate cohort studies with 162,252 participants were eligible for the meta-analysis and 36,253 (22.3%) of them were statin users. Pooled results showed that statin use was independently related to a reduced mortality risk in PLWH [adjusted risk ratio (RR): 0.56, 95% CI: 0.44 to 0.72, *p* < 0.001, *I*^2^ = 41%]. In addition, results of the meta-analysis showed that statin use was not significantly associated with a reduced risk of cardiovascular events in PLWH compared to the statin non-users (RR: 1.14, 95% CI: 0.80 to 1.63, *p* = 0.48, *I*^2^ = 42%). However, statin use was significantly related to a reduced risk of cancer in PLWH (RR: 0.73, 95% CI: 0.58 to 0.93, *p* = 0.009, *I*^2^ = 49%). Sensitivity analyses by excluding one study at a time showed consistent results. No significant publication biases were observed.

**Conclusion:** Statin use is associated with reduced all-cause mortality in PLWH. In addition, statin use is related to a reduced risk of cancer, although the risk of cardiovascular events seems not significantly affected.

## Introduction

People living with HIV (PLWH) have been associated with higher risk of the atherosclerotic cardiovascular diseases (ASCVDs) (1, 2) and non-AIDS-defining cancers (3, 4), which in together with HIV infection itself lead to a higher mortality risk of the population. Pathophysiologically, HIV infection is associated with persistently activated inflammation, which has been recognized as a key factor for the pathogenesis and progression of cardiovascular (CV) events and cancer (5, 6). With the use of lifesaving antiretroviral therapy (ART), the mortality of PLWH has been reduced (7). However, a general life expectancy gap of 8 to 9 years remains between PLWH and general population (8). Therefore, identification of the treatment strategies that benefits clinical prognosis in PLWH remains of great significance.

Statins, also known as 3-hydroxy-3-methylglutaryl CoA (HMG-CoA) reductase inhibitors, are a category of cholesterol-lowering medications, which have become a mainstay for the primary and secondary prevention of ASCVDs (9, 10). In general population with an increased risk of ASCVDs, statin has been associated with reduced risk of CV events, CV mortality, and all-cause mortality (11). Besides, their lipid-lowering property, statins also have pleiotropy of pharmacological potentials such as anti-inflammation, immunomodulation, proapoptosis, antiproliferation, and anti-invasion (12, 13). Accordingly, statin use has been associated with reduced risk of cancer and cancer mortality in general population and people with diabetes (14–16). Moreover, statin use has also been related with reduced CV events and improved survival in the patients with immune-mediated inflammatory diseases (17). In PLWH on ART, statin use has been associated with a reduced risk of HIV rebound (18). In addition, previous meta-analyses have confirmed the efficacy and safety of statins in improving lipid profile in PLWH (19, 20). However, studies evaluating the influence of statin therapy on the risk of CV events, cancer, and all-cause mortality in PLWH showed inconsistent results (21–32). Some studies suggested the benefits of statins on the above outcomes (22, 25, 30, 31), while other studies did not suggest any benefits of statins (21, 23, 24, 26–29, 32). Therefore, we performed a meta-analysis to systematically evaluate the efficacy of statin on the risk of CV events, cancer, and all-cause mortality in PLWH.

## Methods

The instructions of the Meta-analysis of Observational Studies in Epidemiology (MOOSE) (33) and the Cochrane Handbook (34) were followed in the study.

### Literature Search

Studies were obtained by search of the electronic databases of the Medline, the Embase, and the Web of Science databases via the combined search terms: (1) “human immunodeficiency virus” or “human immunedeficiency virus” or “human immune-deficiency virus” or “HIV” or “acquired immune-deficiency syndrome” or “acquired immunedeficiency syndrome” or “acquired immunodeficiency syndrome” OR “acquired immuno-deficiency syndrome” or “AIDS”; (2) “statin” or “3-hydroxy-3-methyl-glutaryl-CoA reductase inhibitor” or “CS-514” or “statin” or “simvastatin” or “atorvastatin” or “fluvastatin” or “lovastatin” or “rosuvastatin” or “pravastatin” or “pitavastatin”; and (3) “death” or “mortality” or “deaths” or “survival” or “cardiovascular” or “stroke” or “coronary” or “myocardial infarction” or “cancer” or “malignancy” or “malignant” or “tumor.” Only studies in human were considered and the publication language was limited to English. The references of the related original and review articles were further screened manually for possible studies. The literature search was finally performed on June 14, 2021.

### Study Selection

The inclusion criteria were: (1) cohort studies; (2) included adult PLWH; (3) statin use was considered as exposure; (4) compared the incidence of at least one of the following outcomes between the users and non-users of stain including all-cause mortality, newly developed CV events, and cancer; and (5) reported the risk ratio (RR) for the association between statin use and the incidence of the above outcomes in the multivariate analysis. The definition of statin use was consistent with the criteria applied among the included studies. The CV events generally included a composite outcome of coronary artery disease (CAD), stroke, peripheral vascular disease, heart failure, and CV deaths. Reviews, duplicated studies, studies not in PLWH, or studies without available outcome data were not included.

### Data Extracting and Quality Evaluation

The processes of database search, data extraction, and study quality assessment were independently and separately performed by the two authors. If discrepancies occurred, a discussion with the corresponding author was indicated. Following data were recorded into a predefined Excel form for data management: (1) name of the first author, publication year, and study location; (2) category of study design; (3) characteristics of the patient such as information regarding the diagnosis, sample size, age, and sex; (4) definition of statin use and numbers of statin users in each study; (5) follow-up durations and outcomes reported; and (6) confounding factors that were adjusted. The Newcastle–Ottawa Scale (NOS) (35) was used for study quality evaluation based on the three domains including group selection of the patients, between-group comparability, and outcome determination. This scale was with a score band of 1 to 9, of which 9 indicates the highest study quality.

### Statistical Analyses

Risk ratios and their corresponding 95% CIs were chosen as the general measure for the relationship between statin use and incidence of all-cause mortality, CV events, and cancer. Data of RRs were extracted directly from the original studies and SEs of RRs were calculated based on 95% CIs or *p*-values reported in the original papers. Data of RRs were further logarithmically transformed to stabilize variance and normalized the distribution (34). The heterogeneity evaluation was achieved by the Cochran's *Q* test and calculation of *I*^2^ statistic (36). A significant heterogeneity was considered if *I*^2^ > 50%. The outcome data were combined with a random-effects model by incorporating the between-study heterogeneity (34). Sensitivity analyses by sequentially excluding one dataset a time were performed to evaluate possible influence of certain study on the outcome (37). The potential publication bias was assessed by visual examination for the symmetry of the funnel plots and the results of the Egger's regression asymmetry test (38). *p* < 0.05 indicates statistically significant. The RevMan (Version 5.1; Cochrane Collaboration, Oxford, UK) software was applied for the meta-analysis and statistics.

## Results

### Literature Search

**Figure 1:**
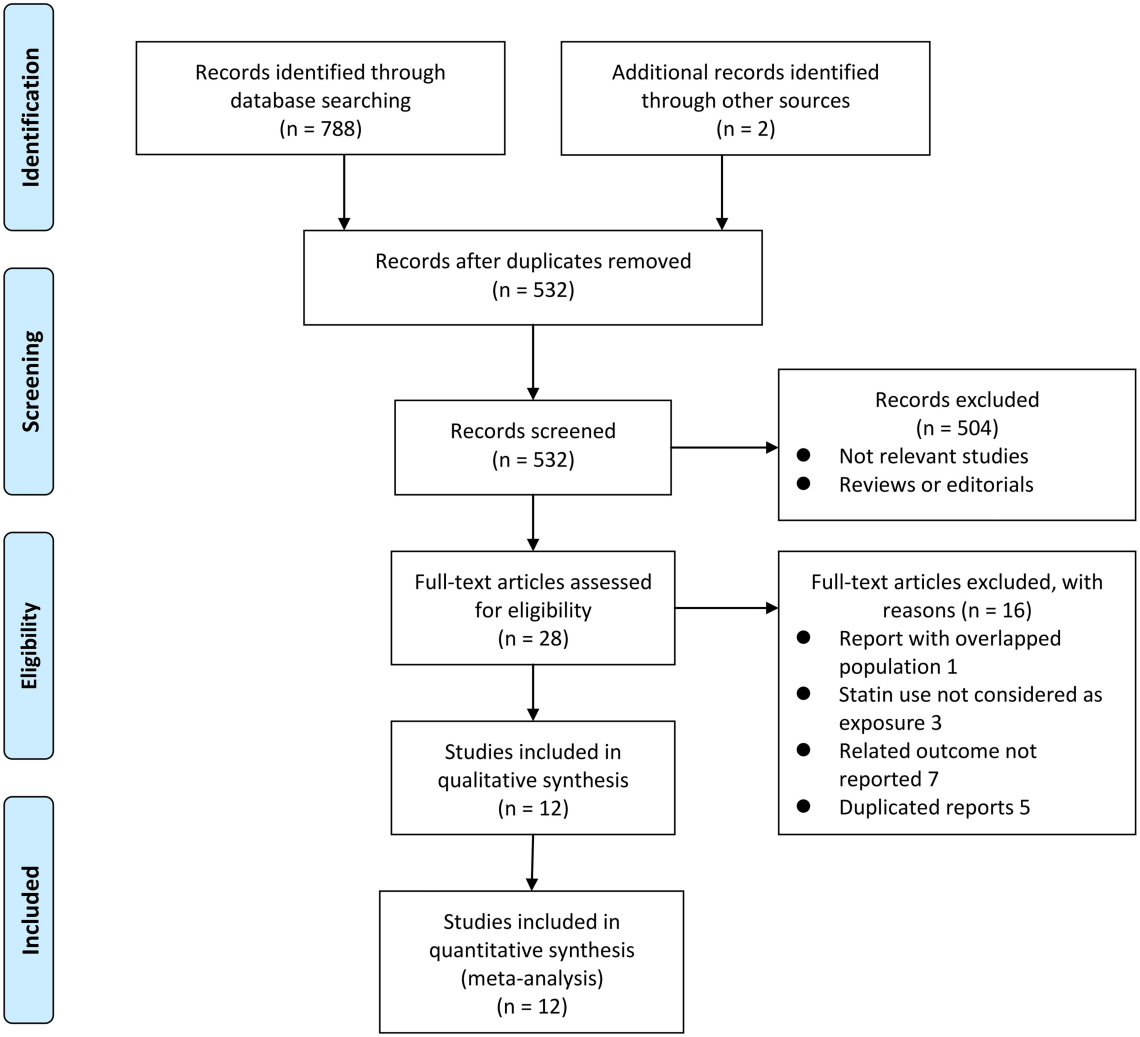
Flowchart of literature search.

Figure 1 shows the flowchart of database search and study identification. It was shown that a total of 5,320 studies were obtained after literature search in the Medline, the Embase, and the Web of Science databases after excluding of the duplicated records, while 504 of them were excluded based on the titles and abstracts primarily because they were irrelevant studies. Then, 16 of the 28 potentially relevant studies were further excluded based on full-text review according to the reasons listed in Figure 1 and the other 12 studies were finally included (21–32).

### Study Characteristics and Quality Evaluation

Table 1 shows the summarized characteristics of the included studies. These studies were all designed as the cohort studies [10 prospective studies (21–25, 27, 28, 30–32) and 2 retrospective studies (26, 29)] including PLWH from the United States, Denmark, Spain, Italy, and France. A total of seven studies included PLWH on ART (21, 23–26, 28, 31), while the others did not specify the concurrent therapy for HIV. The sample sizes of the included studies varied between 127 and 82,426 with a total of 162,252 participants. The mean ages of the PLWH included in each study varied between 39 and 54 years with proportions of the males ranging from 67 to 98%. Generally, PLWH with statin use during follow-up were defined as the statin users and in total, 36,253 (22.3%) of them were the statin users in this meta-analysis. The follow-up duration varied from 1.6 to 10.1 years with a mean follow-up duration of 3.8 years. Outcomes of mortality, CV events, and cancer were reported in nine (21–24, 26–28, 30, 31), five (23, 26, 29, 31, 32), and four (23, 25, 30, 31) studies, respectively. Factors including demographic factors, CV risk factors, comorbidities, and HIV-related factors were adjusted to a varying degree in the multivariate models among the included studies. Table 2 shows the summarized details of study quality evaluation. The NOS scores were 7 to 9 for these studies, which generally reflected the good study quality.

**Table 1:** Characteristics of the included cohort studies.

**Study**	**Country**	**Design**	**Participants**	**Sample size**	**Mean age**	**Men**	**Statin therapy**	**Number of patients with statin therapy**	**Follow-up duration**	**Outcome reported**	**Variables adjusted**
					**Years**	**%**		***n* (%)**	**Years**		
Moore et al. (21)	USA	PC	PLWH on HAART	1,538	43	67.2	Statin use at the time of or after HAART for at least 30 days	238 (15.5)	1.6	All-cause mortality	Age, race, HIV risk group, prior use of ART, CD40, HIV-1 RNA, prior AIDS-defining illness, TC, HGB, and viral hepatitis coinfection
Overton et al. (23)	USA	PC	PLWH on ART	3,601	39	83	Statin use at the time of or after ART	484 (13.4)	4.2	CV events, cancer, and all-cause mortality	Age, sex, race/ethnicity, intravenous drug history, history of CAD, hepatitis B coinfection, systolic BP, eGFR, glucose, current use of lipid-lowering drugs other than statins, HIV-1 RNA, CD4 count, current smoking, and WHR
Rasmussen et al. (24)	Denmark	PC	PLWH on HAART	1,738	39.3	73.1	Statin use at the time of or after HAART	124 (7.1)	4.6	All-cause mortality	Age, sex, race, HIV transmission group, CD4, HIV-RNA, TC, year of HAART initiation, ART prior to HAART, AIDS defining illness prior to HAART initiation, viral hepatitis C co-infection, and comorbidities
Knobel et al. (22)	Spain	PC	PLWH	733	42.1	72.2	Statin use during follow-up	154 (21)	10.1	All-cause mortality	Age, baseline CD4 cell count, baseline HIV-RNA, undetectable viral load at follow-up, Framingham risk score, HIV transmission group, chronic liver disease, and smoking status
Galli et al. (25)	Italy	PC	PLWH on ART	5,357	46.5	76	Statin use at the time of or after ART	740 (14)	9.8	Cancer	Age, sex, smoke, HIV risk factor, HBs-Ag, Ab-anti HCV, CD4, HIV-RNA, TC, TG, FPG, and HGB
Krsak et al. (26)	USA	RC	PLWH on combined ART	438	44	68	Statin use at the time of or after ART	67 (15.3)	5.2	CV events, and all-cause mortality	Age, sex, race, HCV and HBV co-infection, presence of MetS, Framingham risk score percentage, time varying LDL level, baseline HDL level, CD4 count, time from start of ART, smoking status and baseline protease inhibitor use
Lang et al. (27)	France	PC	PLWH	1,776	48.1	89	Statin use during follow-up	138 (8)	4.4	All-cause mortality	Age, sex, HIV transmission group, CD4 T cell count, plasma HIV-1 RNA, anti-HCV Ab and HBs-Ag status, BMI, HGB, smoking, hypertension and diabetes
Rosenson et al. (29)	USA	RC	PLWH	82,426	NR	83.9	Statin use during follow-up	15,619 (18.9)	2	CV events	Age, sex, calendar year, smoking, geographic region of residence, history of CVD, DM, hypertension, CKD, depression, and concurrent medications
Phan et al. (28)	USA	PC	PLWH on HAART	127	54	94	Statin use at the time of or after HAART	28 (22)	3.2	All-cause mortality	Age, sex, race, traditional risk factors, and HIV-related risk factors
Bedimo et al. (30)	USA	PC	PLWH	40,029	52.7	97.1	Newly initiating statin use at least two prescription fills within 180 days	12,153 (30.4)	5.7	Cancer, and all-cause mortality	Age, sex, race, BMI, smoking, HBV, HCV, DM, HIV-RNA, and CD40
Drechsler et al. (31)	USA	PC	PLWH on HAART	23,276	53	97.5	Statin use at the time of or after HAART for ≥11/12 past months	6,458 (27.7)	5.2	CV events, cancer, and all-cause mortality	Age, sex, race, BMI, CD40, HIV viral load, smoking, untreated BP, prevalent CVD, HGB, TC, HDL-C, and eGFR
Tan et al. (32)	France	PC	PLWH with HCV	1,213	45.4	70.3	Statin use during follow-up	50 (4.1)	5.1	CV events	Age, sex, smoking, DM, and arterial hypertension, TC, HDL-C, HIV viral load, and CD40

*PC, prospective cohort; RC, retrospective cohort; PLWH, people living with HIV; HARRT, highly active antiretroviral therapy; ART, antiretroviral therapy; HCV, hepatitis B virus; CV, cardiovascular; BMI, body mass index; DM, diabetes mellitus; TC, total cholesterol; LDL-C, low-density lipoprotein cholesterol; HDL-C, high-density lipoprotein cholesterol; BP, blood pressure; HGB, hemoglobin; eGFR, estimated glomerular filtration rate; CVD, cardiovascular disease; CAD, coronary artery disease; CKD, chronic kidney disease; HBV, hepatitis B virus; FPG, fasting plasma glucose*.

**Table 2:** Details of study quality evaluation via the Newcastle–Ottawa Scale.

**Study**	**Representativeness of the exposed cohort**	**Selection of the non-exposed cohort**	**Ascertainment of exposure**	**Outcome not present at baseline**	**Control for age**	**Control for other confounding factors**	**Assessment of outcome**	**Enough long follow-up duration**	**Adequacy of follow-up of cohorts**	**Total**
Moore et al. (21)	1	1	1	1	0	1	1	0	1	7
Overton et al. (23)	1	1	1	1	1	1	1	1	1	9
Rasmussen et al. (24)	1	1	1	1	1	1	1	1	1	9
Knobel et al. (22)	1	1	1	1	0	1	1	1	1	8
Galli et al. (25)	1	1	1	1	1	1	1	1	1	9
Krsak et al. (26)	0	1	1	1	1	1	1	1	1	8
Lang et al. (27)	1	1	1	1	1	1	1	1	1	9
Rosenson et al. (29)	1	1	1	1	1	1	1	0	1	8
Phan et al. (28)	0	1	1	1	1	1	1	1	1	8
Bedimo et al. (30)	1	1	1	1	1	1	1	1	1	9
Drechsler et al. (31)	1	1	1	1	1	1	1	1	1	9
Tan et al. (32)	0	1	1	1	1	1	1	1	1	8

### Association Between Statin Use and All-Cause Mortality in People Living With HIV

**Figure 2:**
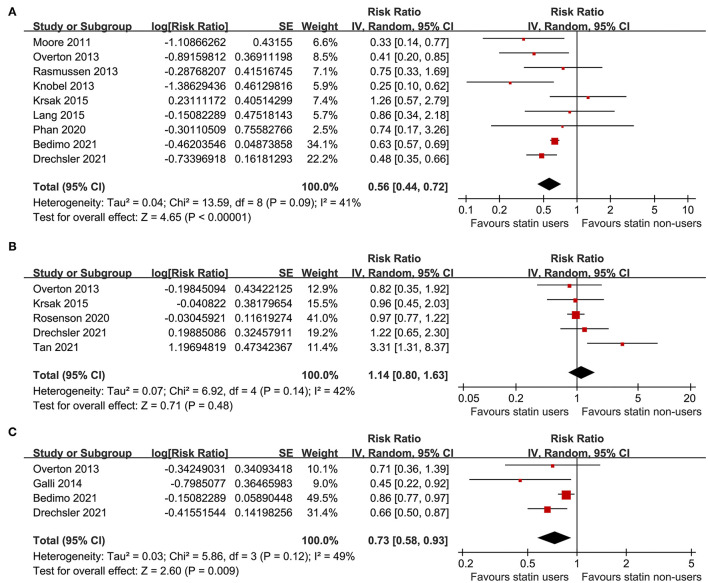
Forest plots for the meta-analyses of the association between statin use and clinical outcomes in people living with HIV (PLWH); **(A)** meta-analysis for the outcome of all-cause mortality; **(B)** meta-analysis for the risk of cardiovascular (CV) events; and **(C)** meta-analysis for the risk of incidence of cancer.

A total of nine studies (21–24, 26–28, 30, 31) including 73,256 PLWH evaluated the association between statin use and all-cause mortality. Pooled results showed that statin use was independently related to a reduced mortality risk in PLWH (adjusted RR: 0.56, 95% CI: 0.44 to 0.72, *p* < 0.001; Figure 2A) with moderate heterogeneity (*p* for Cochran's *Q* test = 0.09, *I*^2^ = 41%). Sensitivity analysis by excluding one study at a time showed the consistent results (RR: 0.54 to 0.59, *p* < 0.05). Specifically, studies limited to the prospective cohort studies also showed the consistent results (eight studies, RR: 0.54, 95% CI: 0.43 to 0.67, *p* < 0.001, *I*^2^ = 33%). In addition, studies limited to PLWH on ART also showed the similar results (six studies, RR: 0.56, 95% CI: 0.39 to 0.80, *p* = 0.001, *I*^2^ = 34%).

### Association Between Statin Use and Risks of Cardiovascular Events and Cancer in People Living With HIV

A total of five studies (23, 26, 29, 31, 32) including 110,954 PLWH evaluated the association between statin use and risk of CV events. Details of the definition of CV events in each of the included studies are shown in Table 3, which are not identical. Pooled results showed that statin use was not significantly related to the risk of CV events in PLWH (RR: 1.14, 95% CI: 0.80 to 1.63, *p* = 0.48, *I*^2^ = 42%; Figure 2B). Sensitivity analysis by excluding one study at a time showed consistent results (RR: 0.98 to 1.25, *p* > 0.05). However, a meta-analysis of the four studies (23, 25, 30, 31) showed that statin use was significantly related to a reduced risk of cancer in PLWH (RR: 0.73, 95% CI: 0.58 to 0.93, *p* = 0.009, *I*^2^ = 49%; Figure 2C). Sensitivity analyses by excluding one study at a time also showed consistent results (RR: 0.64 to 0.78, *p* < 0.05).

**Table 3:** Definition of CV events among the five included studies.

**Studies**	**Definition of CV events**
Overton et al. (23)	Acute myocardial infarction, angina unstable, acute coronary syndrome, myocardial infarction, and silent myocardial infarction Embolic stroke, cerebral thrombosis, thrombotic stroke, central nervous system hemorrhages and cerebrovascular accidents, carotid artery stenosis, transient ischemic attack, cerebrovascular and spinal necrosis and vascular insufficiency Angioplasty, coronary angioplasty, coronary arterial stent insertion, coronary revascularization, coronary artery bypass, and coronary endarterectomy
Krsak et al. (26)	Myocardial infarction, stroke, or cardiovascular mortality
Rosenson et al. (29)	Myocardial infarction, stroke, and low extremity artery disease hospitalizations
Drechsler et al. (31)	Coronary events, cerebrovascular events, or cardiovascular mortality
Tan et al. (32)	Coronary events, cerebrovascular events, peripheral artery disease, or cardiovascular mortality

### Publication Bias

**Figure 3:**
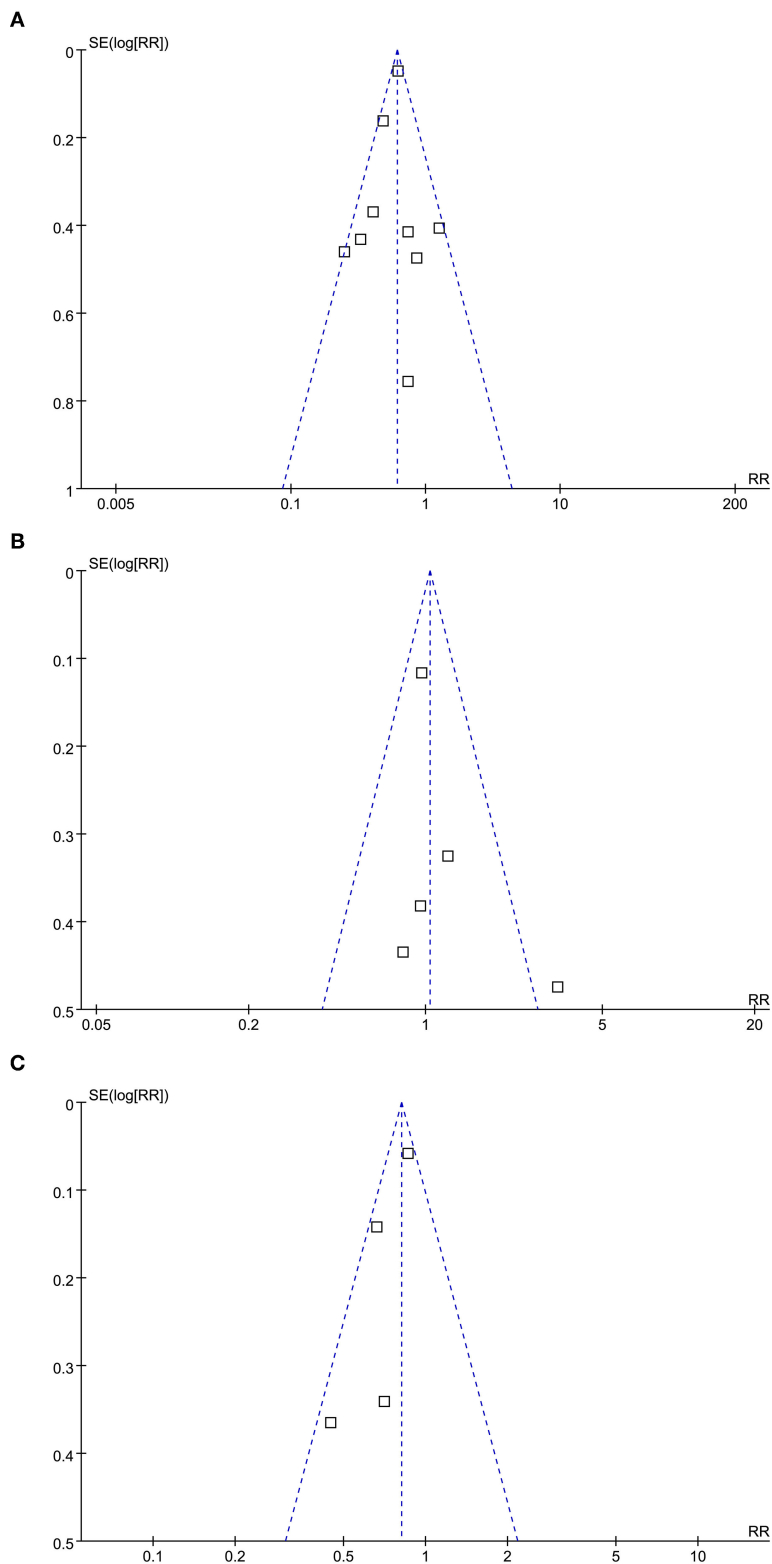
Funnel plots for the meta-analyses of the association between statin use and clinical outcomes in PLWH; **(A)** funnel plots for the meta-analysis of all-cause mortality; **(B)** funnel plots for the meta-analysis of CV events; and **(C)** funnel plots for the meta-analysis of incidence of cancer.

The funnel plots for the meta-analyses of the association between statin use and mortality, CV events, and cancer risks are shown in Figures 3A–C. The plots were symmetrical on visual inspection, suggesting a low risk of the publication biases. Results of the Egger's regression tests also suggested low risks of publication bias for the meta-analysis between statin use and mortality risk (*p* = 0.522). For the meta-analyses of CV events and risks of cancer, the Egger's regression tests were not performed, since the limited numbers of datasets were included.

## Discussion

In this meta-analysis of the cohort studies, we found that in PLWH, statin use was significantly related to a reduced risk of all-cause mortality and a decreased incidence of cancer. However, the incidence of CV events was not significantly affected. These results suggested that although previous clinical trials have proved the benefits of statin in improving lipid profile in PLWH, current evidence from the epidemiological studies did not show that statin use was associated with a reduced risk of CV events in this population. However, statin use in PLWH is associated with improved survival and according to our results, reducing cancer-related adverse events are likely to be involved in the benefits of statin in PLWH.

As far as we know, only one previous meta-analysis published in 2018 evaluated the association between statin use and all-cause mortality in PLWH (39). This meta-analysis included seven cohort studies and concluded that statin use may be associated with improved survival in PLWH (39). Although the conclusion of our meta-analysis is similar to the previous one, this study has several additional strengths. Firstly, the current meta-analysis is the largest meta-analysis of a similar topic to date which integrated the up-to-date evidence regarding the influence of statin on mortality in PLWH. The numbers of included studies (12 vs. 7) and participants (162,252 vs. 35,708) in this meta-analysis are much larger than that of the previous one (39). Secondly, the robustness of the findings was evidenced by the results of sensitivity analyses by excluding one study at a time, which was not performed in the previous meta-analysis. Moreover, since the use of ART was associated with the severity of dyslipidemia and subsequent risk of CVD (40, 41), we performed a sensitivity analysis including only studies with PLWH on ART and a consistent benefit of statin on survival outcome in these patients was observed. In addition, we also performed sensitivity analysis limited to the studies with prospective cohort design to avoid the possible selection and recall biases inherited with retrospective studies and a consistent result was also observed. Finally, besides the outcome of all-cause deaths, we also observed the associations between statin use with CV events and incidence of cancer in PLWH, which to the best of our knowledge, has not been evaluated in the previous meta-analysis.

Surprisingly, we found that the use of statin was not significantly associated with a reduced risk of CV events in PLWH. Although only five studies were included, the results seemed stable because none of the included studies showed an inverse association between statin use and risk of CV events. Several explanations may be helpful to understand the results. The most likely one is that although statins are effective in reducing total cholesterol (TC) and low-density lipoprotein cholesterol (LDL-C) in PLWH as shown in the previous clinical trials (3, 19), the use of statin is just not adequate to prevent CV events in PLWH considering that the multiple risk factors are involved in the pathogenesis of CVD in PLWH besides increased cholesterol such as persistent inflammatory response, immune cell activation, immunodeficiency, coinfection of *cytomegalovirus*, and mechanisms related to ART (42). The other possible mechanisms may be the bias caused by the indications. Statins are underutilized in PLWH and are generally prescribed in the patients with high CV risk. Accordingly, the imbalance of CV risk between the users and non-users of statins in PLWH may bias the results. However, the possibility is low since conventional CV risk factors were generally adjusted in the multivariate analysis when the association between statin and risk of CV events were evaluated. Moreover, the definitions of CV events were not identical among the five included studies and a different mixture of these CV events may have affected the different responses in these single studies. From this perspective, clinical trials are important for the determination of the influence of statins on the CV events in PLWH (43). Another interesting finding of the meta-analysis is that statin use is significantly associated with a reduced incidence of cancer in PLWH. These results are consistent with the findings in general population without HIV infection (44, 45). Since cancer-related deaths have become an important cause of mortality in PLWH, particularly in those receiving highly active ART (HAART) (46, 47), the possible mechanisms and efficacy of statin use in the prevention of cancer in PLWH deserve further investigation.

This study has some limitations. Firstly, one important limitation of this meta-analysis is the nature of the exposure studied. Among the included observational studies, only the study by Bedimo et al. (30) explicitly declared a “new-user design” approach. In the other studies, statins were administered “during follow-up” or “at the time or after HAART” through a “prevalent user” design approach, which is very prone to selection bias as the start of the follow-up and the start of the intervention do not coincide for all the participants. This may significantly affect the reliability of the findings. Therefore, the results of the meta-analysis should be considered as hypothesis generating and clinical trials are warranted to validate these findings. Secondly, since a comparison between the individual categories of statin medication was not reported among the included studies, a subgroup analysis according to the individual drug used could not be performed. This is important for the selection of the optimized statin for PLWH considering the possible interaction between statins and ART (48). Moreover, influences of dose and duration of statin on the association between statin use and survival outcome in PLWH could not be analyzed. Further studies are needed in the future, since it is important to determine the optimal regimen for statin treatment in these patients. In addition, since this is a meta-analysis based on the data of the study level, we were unable to determine whether the association between statin use and survival in PLWH could be affected by the patient or tumor characteristics such as age, ethnicity, comorbidities of the patients, and concurrent medications. Meta-analysis based on the individual–patient data may be considered for further evaluation. Besides, a protocol has not been published. However, the meta-analysis was strictly performed and reported according to the predefined criteria. Furthermore, the literature research was carried out only on the three electronic databases. Gray literature sources were not explored, no personal communication with the authors of the studies was conducted to the recovery of material never published, and studies published in the other languages, besides English, were not considered. The formal analysis of the presence of publication bias performed by visual inspection (funnel plot) and with formal statistical tests does not guarantee the absence of publication bias due to the small number of trials included in the meta-analytic outcome of an individual. Finally, although studies with the multivariate analysis were included, there could be residual factors that confound the association between statins and clinical outcomes in PLWH.

In conclusion, the results of this meta-analysis showed that statin use was significantly related to a reduced risk of all-cause mortality and a decreased incidence of cancer in PLWH. However, the incidence of CV events was not significantly affected. Although clinical trials are needed to validate the findings, these results suggested that statin use in PLWH is associated with improved survival and reducing cancer-related adverse events is likely to be involved in the benefits of statin in PLWH.

## Data Availability Statement

The original contributions presented in the study are included in the article/supplementary material, further inquiries can be directed to the corresponding author/s.

## Author Contributions

YL and ZW conceived and designed the study. YL and HX selected the studies and collected the data. YL and JZ analyzed the data. YL drafted the manuscript. All the authors interpreted the results, revised the draft manuscript, and read and approved the final version of the manuscript.

## Funding

This study was supported by the National Natural Science Foundation of China (No. 81904040).

## Conflict of Interest

The authors declare that the research was conducted in the absence of any commercial or financial relationships that could be construed as a potential conflict of interest.

## Publisher's Note

All claims expressed in this article are solely those of the authors and do not necessarily represent those of their affiliated organizations, or those of the publisher, the editors and the reviewers. Any product that may be evaluated in this article, or claim that may be made by its manufacturer, is not guaranteed or endorsed by the publisher.
